# Concentration Retrieval in a Calibration-Free Wavelength Modulation Spectroscopy System Using Particle Swarm Optimization Algorithm

**DOI:** 10.3390/s23146374

**Published:** 2023-07-13

**Authors:** Tingting Zhang, Yongjie Sun, Pengpeng Wang, Cunguang Zhu

**Affiliations:** 1 School of Physics Science and Information Technology, Liaocheng University, Liaocheng 252000, China; 2 School of Physics and Technology, University of Jinan, Jinan 250022, China

**Keywords:** particle swarm optimization algorithm, wavelength modulated spectroscopy, calibration-free, spectral fitting

## Abstract

This paper develops a concentration retrieval technique based on the particle swarm optimization (PSO) algorithm, which is used for a calibration-free wavelength modulation spectroscopy system. As compared with the commonly used technique based on the Levenberg–Marquardt (LM) algorithm, the PSO-based method is less dependent on the pre-characterization of the laser tuning parameters. We analyzed the key parameters affecting the performance of the PSO-based technique and determined their optimal parameter values through testing. Furthermore, we conducted a comparative analysis of the efficacy of two techniques in detecting C_2_H_2_ concentration. The results showed that the PSO-based concentration retrieval technique is about 63 times faster than the LM-based one in achieving the same accuracy. Within 5 s, the PSO-based technique can produce findings that are generally consistent with the values anticipated.

## 1. Introduction

Wavelength modulation spectroscopy (WMS) technology has been extensively used to measure gas characteristics in areas such as greenhouse gas and atmospheric pollutant monitoring [1,2], combustion monitoring [3,4], and industrial process control [5,6,7] due to its noise rejection, high measurement accuracy, simplicity, and fast response time [8,9,10,11,12,13,14].

In early applications, WMS requires calibration with a gas mixture of known concentrations, which limits the utilization of WMS technology in harsh environments (such as high temperature or pressure) or where the relevant gas conditions are poorly known. Thus, researchers have developed numerous calibration-free techniques for this issue [15,16,17,18,19,20,21,22]. Li et al. [23] proposed a calibration-free technique combining laser tuning parameters with a WMS analytical model of the absorption spectrum, which can eliminate the majority of calibration factors and infer gas parameters. Rieker et al. [24] utilized the first harmonics to normalize the second harmonics (WMS-2*f*/1*f*) and extended the WMS analysis model to the field of combustion monitoring to retrieve the temperature and concentration of water vapor simultaneously. A gas concentration retrieval strategy based on the first harmonic phase angle (*θ*_1*f*_) method, which is resistant to the laser intensity and the demodulation phase, was proposed by Yang et al. [25]. In order to identify trace gases with no background interference and immunity to changes in light intensity, a second harmonic phase angle method (*θ*_2*f*_) based on WMS was proposed [26]. In the past few decades of calibration-free WMS method development, the Levenberg–Marquardt (LM) algorithm has been widely used to deal with nonlinear least-squares problems in spectral fitting for gas concentration retrieval. Using a method that does not require prior knowledge of the transition line-shape parameters, Christopher et al. [27] established a method that enables precise calibration-free WMS measurements of gas characteristics. Combining the LM algorithm with dual-spectroscopy techniques, Li et al. [28] developed a mid-infrared laser trace gas sensor capable of online monitoring the concentration of multi-component gases. Raza et al. [29] utilized the LM algorithm for least-squares fitting of simulated and measured scanned-WMS-2*f*/1*f* spectra to concurrently monitor information on temperature and concentration changes in CO and NH_3_ in high-temperature environments.

The LM algorithm has been extensively used to assess the gas conditions of calibration-free WMS systems [27,29,30]. It is worth emphasizing that the fitting by the LM algorithm is often unsatisfactory when models with multiple free parameters are encountered. The updated equations of the free parameters in the LM algorithm are Jacobian functions, in which the coefficient matrix is closely related to the computational dimensionality of the algorithm. When the number of free parameters increases, the associated Jacobian coefficient matrix becomes increasingly intricate, thereby leading to a notable impact on both the accuracy and efficiency of the LM algorithm. Meanwhile, it may also let the LM algorithm fall into a local optimum. By employing the pre-characterization with laser tuning parameters in the WMS models, which reduces the number of free parameters, the problem can be avoided. However, this undoubtedly adds the cost and complexity of the testing process. At the same time, it may cause a series of secondary problems, such as measurement errors caused by the failure of the characterized values during the long operation of the instrument.

In this paper, we propose a concentration retrieval technique based on the particle swarm optimization (PSO) algorithm, which is used for a calibration-free wavelength modulation spectroscopy system. Contrasted with the technique based on the LM algorithm, this technique is relatively weakly dependent on the pre-characterization of the laser tuning parameters. For the target gas C_2_H_2_, we evaluate the PSO-based technique and LM-based technique (abbreviated as the PSO technique and LM technique) comparatively by simulation. The findings demonstrated that, notably in the multi-parameter model without exact characterization, the PSO technique performs better in terms of convergence speed and fitting accuracy.

## 2. Theory and Methodology

The WMS-2*f*/1*f* is the most representative calibration-free system. In this study, we will evaluate the performance of the PSO algorithm based on the WMS-2*f*/1*f* system, and in this section, we will first briefly describe the theory of the WMS-2*f*/1*f* system, and then introduce the idea and process of the PSO algorithm to implement the spectral line fitting in this system.

### 2.1. Theory of WMS-2f/1f

In wavelength modulation spectroscopy, the output of the laser is modulated periodically by varying the current injection to produce modulated light intensity *I*_0_(*t*) and modulated light frequency *ν*(*t*):

(1) $$I_{0}(t)=\bar{I_{0}}(t)\left[1+i_{1}\cos(\omega t+\psi_{1})+i_{2}\cos(2\omega t+\psi_{2})\right]$$

(2) $$v(t)={\bar{v}}_{c}(t)+\Delta v\cdot \cos(\omega t)$$

where *I*_0_(*t*) and *ν_c_*(*t*) are the average light intensity and the center frequency, respectively, *i*_1_ and *i*_2_ are the intensity amplitudes of the first- and second-order (normalized by *I*_0_), respectively, modulation angular frequency *ω* = 2*πf_m_*, *f_m_* is the laser modulation frequency, *ψ*_1_ is the phase shift between frequency modulation (FM) and intensity modulation (IM), *ψ*_2_ is the phase shift of the nonlinear IM, Δ*ν* is the modulation depth.

According to the Beer–Lambert law, the transmitted laser intensity at frequency *ν* can be expressed as:

(3) $$I_{t}(t)=I_{0}(t)\cdot \exp\left[-\alpha (v(t))\right]$$

where *I*_0_(*t*) is incident laser intensity, *I_t_*(*t*) is the transmitted light intensity, *α*(*ν*(*t*)) denotes the spectral absorbance.

(4) $$\alpha (v(t))=PS(T)CLg(v,v_{0})$$

where *P* is the total gas pressure, *S*(*T*) is the intensity of the absorption spectrum at *T* temperature, *C* is the concentration of the gas to be measured, *L* is the length of the optical range, the line shape function *g*(*ν*, *ν*_0_) at atmospheric pressure can be described by a Lorentz profile, and it can be written as follows:

(5) $$g(v,v_{0})=\frac{2}{\pi \Delta v_{c}}\frac{1}{1+{\left[x+m\cos(\omega t)\right]}^{2}}$$

(6) $$x=2\frac{v_{c}-v_{0}}{\Delta v_{c}}$$

(7) $$m=\frac{2\Delta v}{\Delta v_{c}}$$

where Δ*ν_c_* is the full width at half-maximum, *ν*_0_ is the line-center frequency of the absorption spectrum, *x* is the normalized frequency, *m* is the modulation index.

Expansion of exp[−*α*(*ν*(*t*))] in a Fourier cosine series under sinusoidal injection current modulation of the laser is as follows:

(8) $$\exp\left[-\alpha (v(t))\right]=\sum_{k=0}^{\infty }H_{k}(v_{c},\Delta v)\cdot \cos(k\omega t)$$

The *I_t_*(*t*) signal is input into the lock-in amplifier (LIA) and multiplied with a reference cosine wave and sine wave at *nf* to extract the *Xnf* and *Ynf* signals, respectively, which are subsequently low-pass filtered to determine *S*_2*f*/1*f*_ [31], given by Equation (9):

(9) $$S_{2f/1f}=\sqrt{{\left(\frac{X_{2f}}{X_{1f}}\right)}^{2}+{\left(\frac{Y_{2f}}{Y_{1f}}\right)}^{2}}\;=\sqrt{{\left(\frac{H_{2}+\frac{i_{1}}{2}(H_{1}+H_{3})\cos\psi_{1}+i_{2}H_{0}\cos\psi_{2}}{H_{1}+i_{1}(H_{0}+\frac{H_{2}}{2})\cos\psi_{1}+\frac{i_{2}}{2}(H_{1}+H_{3})\cos\psi_{2}}\right)}^{2}+{\left(\frac{\frac{i_{1}}{2}(H_{1}-H_{3})\sin\psi_{1}+i_{2}H_{0}\sin\psi_{2}}{i_{1}(H_{0}-\frac{H_{2}}{2})\sin\psi_{1}+\frac{i_{2}}{2}(H_{1}-H_{3})\sin\psi_{2}}\right)}^{2}}$$

From Equation (9), it is clear that the spectral model *S*_2*f*/1*f*_ is a function of multiple parameters, including the modulation index *m*, gas concentration *C*, the first- and second-order mean-intensity-normalized intensity amplitudes *i*_1_ and *i*_2_, and the phase shifts *ψ*_1_ and *ψ*_2_ between FM and linear and nonlinear IM, among other parameters. This presents a significant obstacle for the LM technique, which we shall successfully resolve using the PSO technique.

### 2.2. PSO-Based WMS Concentration Retrieval Technique

The PSO algorithm is an evolutionary computing method based on swarm intelligence that mimics the social interaction and migration of bird flocks, as well as the social sharing of knowledge to aid in the evolution of individuals. Its core idea is to use the in-formation interaction among a population of particles to seek the optimal solution to a multi-parameter optimization problem. In this paper, we apply it to a multi-parameter optimization of the spectral model *S*_2*f*/1*f*_ for gas concentration and laser parameter retrieval in a calibration-free WMS system.

In the proposed technique, the simulated spectra are fitted to the measured spectra. The concentration is obtained from the optimal solution of the fitting, which error is determined by the objective function *F*(*η_id_*):

(10) $$F(\eta_{id})=\sum_{n=1}^{N}{\left(f_{i}(x_{n},\eta_{id})-y_{n}\right)}^{2}$$

where *x_n_* is the normalized frequency, *n* = 1, 2, 3, …, *N*, *N* is the total number of sampling points in the simulated spectra, *f_i_*(*x_n_*, *η_id_*) is simulated spectral data given by Equation (9), the free parameters are denoted by the vector *η* that defines the current position of the particle, *D* is dependent on the number of free parameters (*d* = 1, 2, 3, …, *D*), *K* is the population of particle (*i* = 1, 2, 3, …, *K*), *y_n_* is the measured spectra.

**Figure 1:**
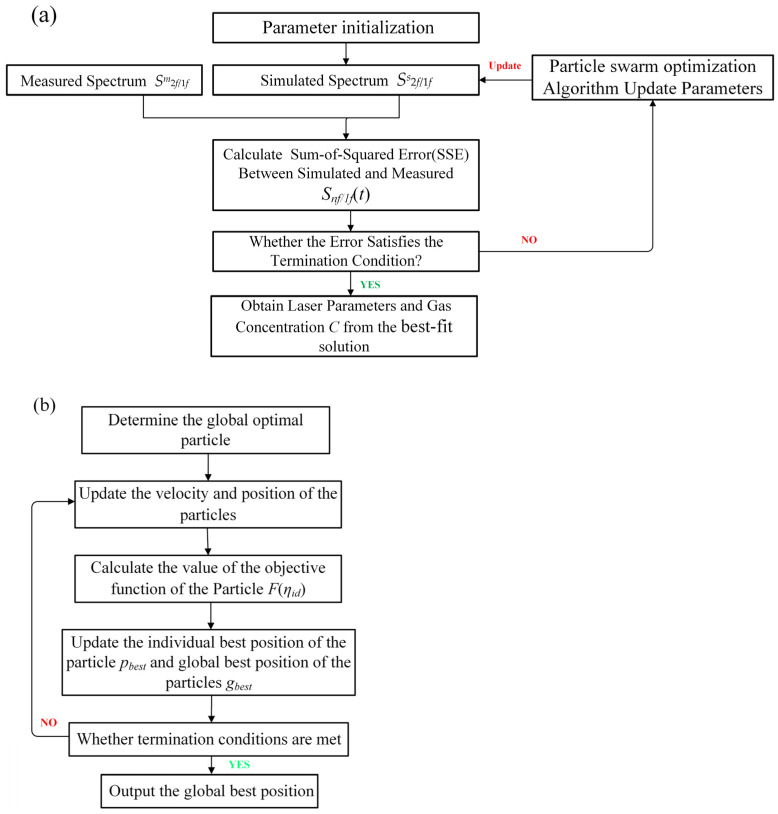
(**a**) Flow chart for seeking the optimal solution of gas properties and laser parameters using the PSO technique. (**b**) The flow chart of PSO technique update parameters.

The PSO-based calibration-free WMS spectral fitting technique flow chart is shown in Figure 1. In this technique, the following parameters can all be set to free parameters of fitting, including the modulation index *m*, gas concentration *C*, the first- and second-order intensity amplitudes *i*_1_ and *i*_2_, and the phase shifts *ψ*_1_ and *ψ*_2_.

Step 1: Parameter initialization—Generate *K* vectors *η_i_* for the fitting procedure, which are randomly assigned within a specific range, *i* = 1, 2, …, *K*;

Step 2: Acquisition of simulated spectra—The simulated spectra *f_i_*(*x_n_*, *η_id_*) are obtained by substituting the initialized parameters *η_i_* into Equation (9);

Step 3: Parameter updating—The objective function *F* of Equation (10) is used to update the fitness values of each particle, which measures the closeness of the corresponding solution with the optimal solution;

Step 4: Judgment—If the convergence between measured spectra *S^m^*_2*f*/1*f*_ does not satisfy the termination condition of the optimization, then continue to Step 5; Otherwise, perform Step 6;

Step 5: Particle movement—Particles rely on velocity and position updates to complete optimization, which determines the search route of particles in space. The position of the particle *i* in the (*t* + 1)th iteration is updated by the following equation:

(11) $$\eta_{id}^{t+1}=\eta_{id}^{t}+v_{id}^{t+1}$$

where *η^t^_id_* is the position of the particle *i* in the *t*th iteration, the velocity of the particle *i* in the (*t* + 1)th iteration *v^t^*^+1^*_id_* can be defined as:

(12) $$v_{id}^{t+1}=\omega \cdot v_{id}^{t}+c_{1}rand()\left(p_{best}^{t}-\eta_{id}^{t}\right)+c_{2}rand()\left(g_{best}^{t}-\eta_{id}^{t}\right)$$

where *v^t^_id_* is the velocity of the particle *i* of *t*th iteration, *ω* ∈ [0,1] is the inertia weight, rand() ∈ [0,1] is a random value, *p^t^_best_* is individual best position of the particle *i* in the *t*th iteration, *g^t^_best_* is the global best position of the any particle in the *t*th iteration, *c*_1_ and *c*_2_ are the real acceleration coefficients that control how much the global and individual best positions should influence the velocity of the particle.

After updating of each particle position and fitness value in Steps 3 and 5, the swarm of particles will keep moving closer to the region of the optimal solution;

Step 6: Termination condition—If the convergence between measured spectra *S^m^*_2*f*/1*f*_ and simulated spectra *S^s^*_2*f*/1*f*_ satisfies the termination condition of the optimization, the value of the best-fit solution is output at this point, and laser parameters and gas concentration *C* are obtained.

## 3. Simulation Verification

The feasibility of the PSO-based WMS spectral fitting technique is verified in the following. In order to avoid the characterization errors introduced by the metrology instruments affecting the evaluation of the algorithm performance, we chose to perform the validation by simulation in the MATLAB R2019b platform (the computer had the following specifications: CPU: i5-10400F, RAM: 8 GB).

The P(13) spectral line of acetylene gas at 1532.83 nm was chosen as the target spectral line. The spectrum parameters used are taken from the HITRAN2016 database. [32] Given the values of the spectral parameters in Table 1. In Equations (1) and (2), *I*_0_(*t*), *ν_c_*(*t*), *I*_0_(*t*), and *v*(*t*) all vary with time. For simplicity of handling, we set both *i*_1_ and *i*_2_ in the program to fixed values. The measured spectra that should have been collected in the experiment were replaced by the simulated data set calculated by Equation (9):

(13) $$y_{n}=\left[\left(x_{1},y_{1}),(x_{2},y_{2}),\ldots ,(x_{4000},y_{4000}\right)\right]$$

**Figure 2:**
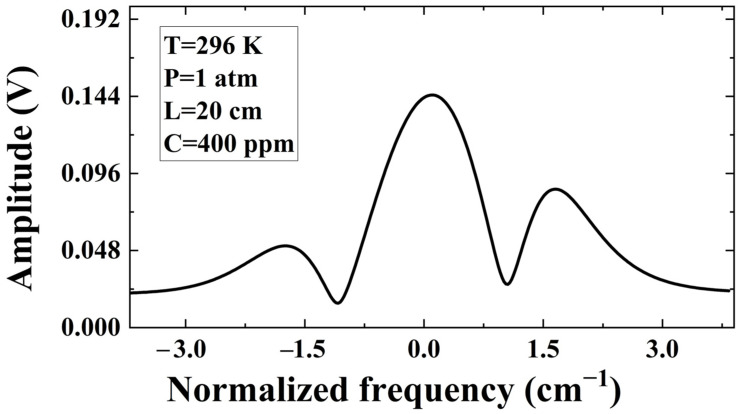
The amplitude profiles of acetylene under the conditions of *C* = 400 ppm, *T* = 296 K, *P* = 1 atm, *L* = 20 cm.

The profile is shown in Figure 2.

The performance of the PSO algorithm is affected by the number of particles and the range of free parameters. Increasing the number of particles can enhance the accuracy of search, but it also leads to a slower convergence rate. Additionally, the search scope of particles is determined by parameter range, and a more precise range allows the algorithm to discover the global optimal solution earlier. The range of free parameters and the number of particles in the simulation are shown in Table 2.

Inertial weight and acceleration coefficient are critical parameters of the PSO technique, and we first meticulously examine the effects of these two parameters on the technique’s performance. In the fitting, the modulation index *m*, gas concentration *C*, the linear and nonlinear IM depth *i*_1_ and *i*_2_, and the phase shift *ψ*_1_ and *ψ*_2_ between FM and linear and nonlinear IM are considered as free parameters.

**Figure 3:**
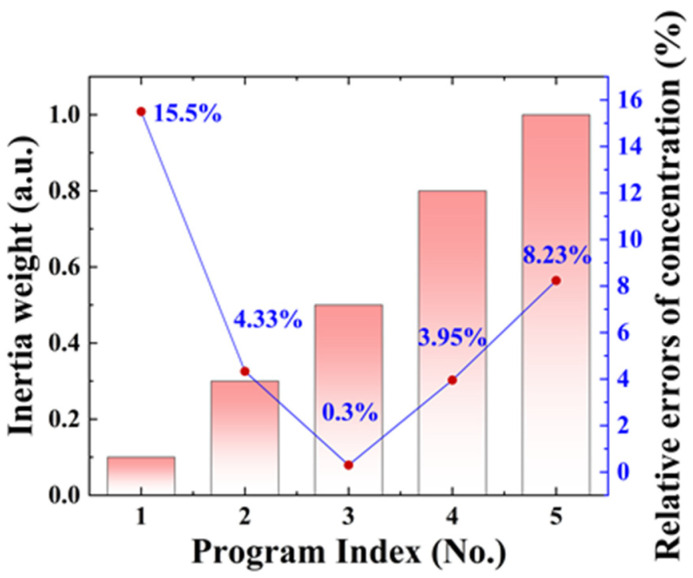
The relative errors of concentration predicted by the PSO technique under different inertia weights.

Inertia weight: The introduction of the inertia weight *ω* helped promote better searching by balancing the global search and the local search. Larger values of *ω* facilitate global exploration, and smaller values promote local exploitation. The suitable value of *ω* results in fewer iterations on average to find a sufficiently optimal solution. Thus, it is crucial to alter the values of the inertia weights to enhance the PSO technique’s performance. Figure 3 shows the fitting results of concentration obtained by varying inertia weights *ω* and keeping acceleration coefficients *c*_1_ = *c*_2_ = 2. From Figure 3, it can be seen that the inertia weight has a great influence on the fitting results, and the relative error of the concentration is the smallest when *ω* is 0.5. Therefore, we set *ω* to 0.5 in the following simulation verification.

**Figure 4:**
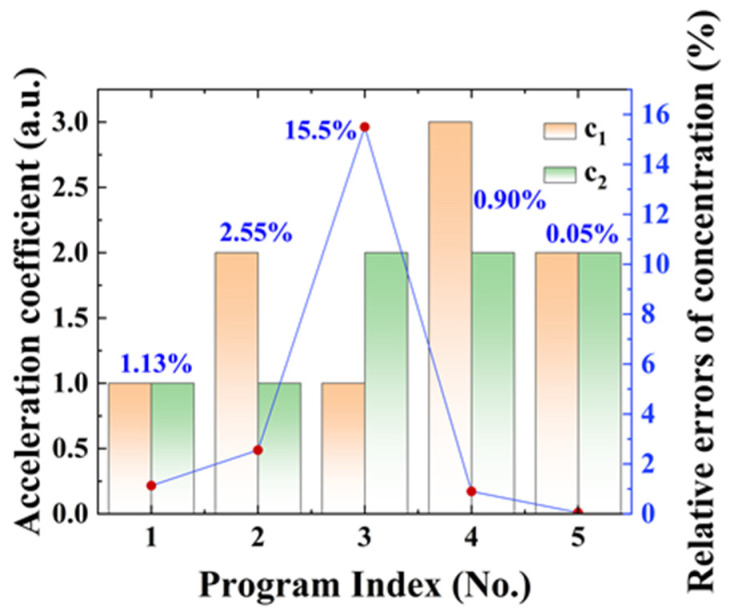
The relative errors of concentration predicted by the PSO technique under different acceleration coefficients.

Acceleration coefficient: *c*_1_ and *c*_2_ are the acceleration coefficients that control how much the global and individual best positions should influence the particle’s velocity. A balance between the values of *c*_1_ and *c*_2_ is required, as inappropriate values of *c*_1_ and *c*_2_ may result in divergent and cyclic behavior of the whole swarm. Figure 4 shows the results of concentration obtained by varying acceleration coefficients *c*_1_ and *c*_2_ and keeping inertia weights *ω* = 0.5. The relative error in concentration is minimal when *c*_1_ = *c*_2_ = 2, as shown in Figure 4. The acceleration coefficients have a substantial impact on the outcomes of the fitting. As a result, we set *c*_1_ and *c*_2_ to 2 in the following simulation validation.

**Figure 5:**
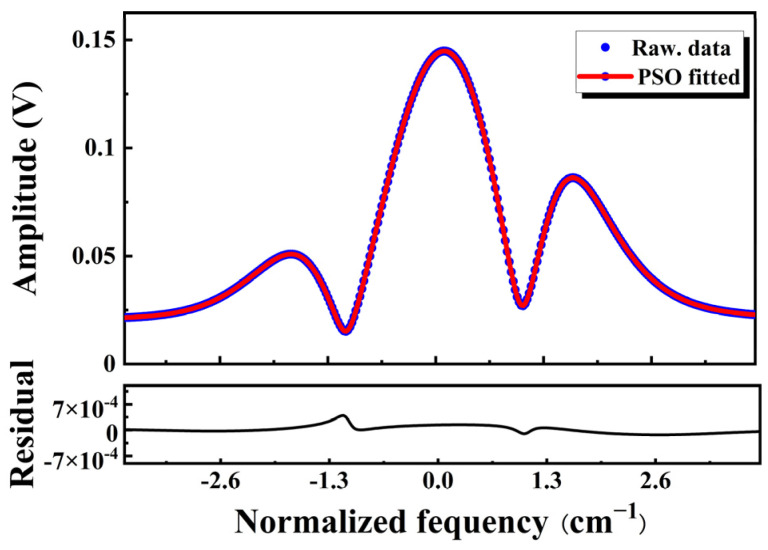
Fitting effect of the PSO technique with a convergence time of 1.6 s and a fitting parameter of *η*_2_ = [*m*, *C*, *i*_1_, *i*_2_, *ψ*_1_, *ψ*_2_].

Figure 5 shows the results of the PSO technique fit the virtual measured spectrum *y_n_* at *ω* = 0.5 and *c*_1_ = *c*_2_ = 2. In this Figure, the PSO technique takes only 1.6 s to bring the residuals to 10^−4^ orders of magnitude. Increasing the convergence time to 5 s results in a significant improvement of the fitting accuracy, as evidenced by the best-fit free parameters indicated in Table 3. Notably, the relative errors of free parameters are less than 1%, which indicates high reliability and accuracy. Furthermore, the predicted values are approximately equal to the expected values, affirming the advantages of this approach.

**Figure 6:**
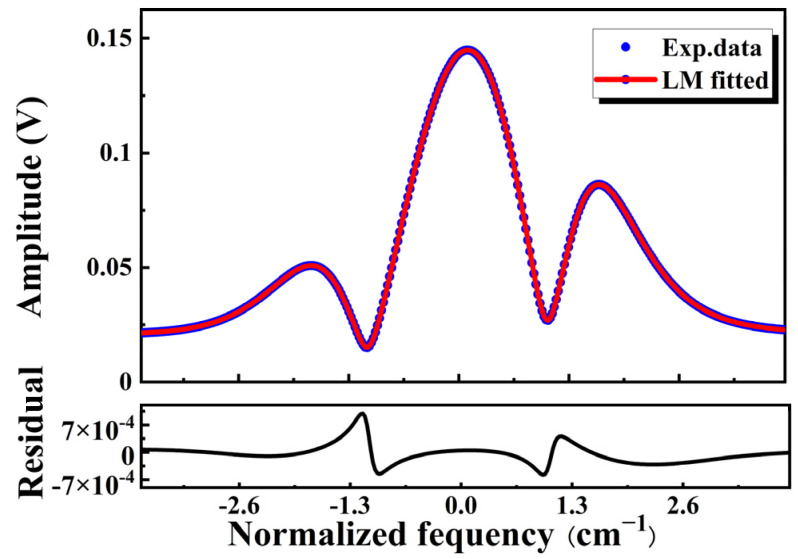
Fitting effect of the LM technique with a convergence time of 100 s and a fitting parameter of *η*_2_= [*m*, *C*, *i*_1_, *i*_2_, *ψ*_1_, *ψ*_2_].

To comprehensively illustrate the effectiveness of the PSO technique in multi-parameter model fitting, we compare it with the LM technique. Specifically, we fit the virtual measurement spectrum *y_n_* by using the LM technique while keeping the same objective function threshold as the PSO technique. The design of initial free parameter values is shown in Table 4, and results are shown in Figure 6. The output best-fit parameters at this point are shown in Table 5. It is clear that the relative errors predicted by the LM technique for the free parameters, such as the linear and nonlinear IM depth *i*_1_ and *i*_2_, and the phase shift *ψ*_1_, exceed 5%.

Comparing Table 2 and Table 4, it is clear that the initial parameter values for the LM technique are designed to be closer to the expected values compared to the PSO technique. However, the final fit of the PSO technique is better, which reflects the good convergence of the PSO technique. According to the results of the above simulation, the PSO-based concentration retrieval technique has a faster convergence rate than the LM technique by a factor of roughly 63 while maintaining residuals at 10^−4^. The fitting relative errors of the PSO technique is significantly improved compared to the LM technique.

**Figure 7:**
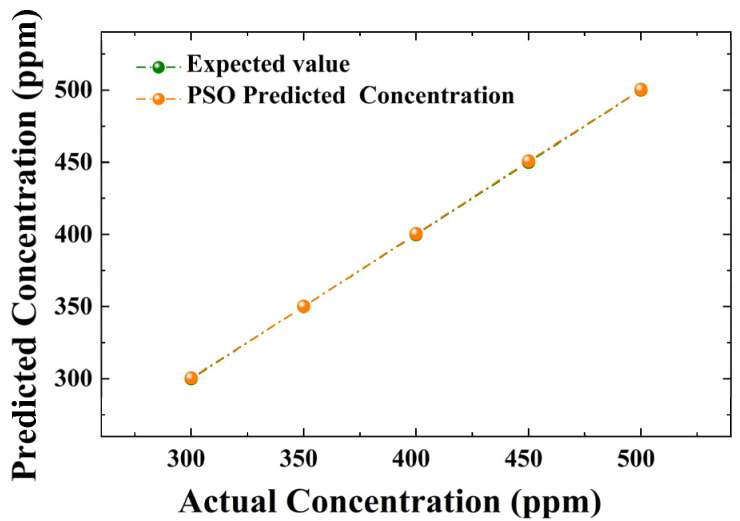
Relationship between expected and predicted concentrations.

**Figure 8:**
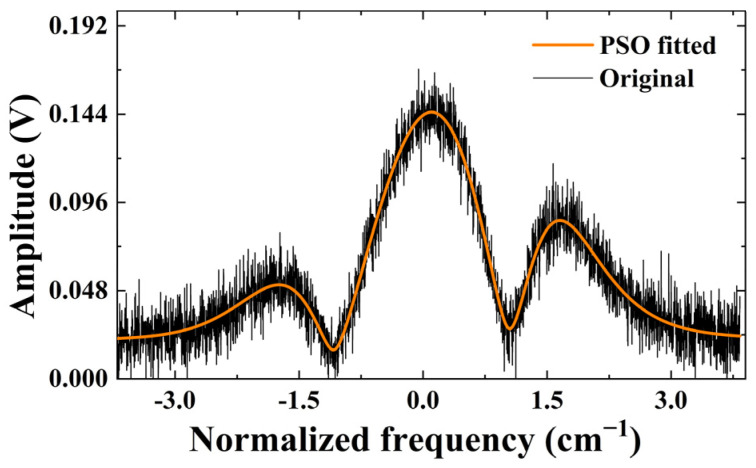
Fitting effect of PSO technique on virtual data containing noise.

In order to ascertain the stability of the PSO technique for monitoring various concentrations, we conducted an analysis at concentrations ranging from 300 to 500 ppm. As depicted in Figure 7, the observed linear relationship between the actual and predicted concentrations demonstrates the technique’s adaptability. In addition, we added noise to test the noise immunity of the PSO technique. We added Gaussian white noise to the original data, and the fitting results are shown in Figure 8. It can be seen that a relatively good fit is still achieved in the presence of noise.

As one of the metaheuristics, the PSO algorithm also has a certain chance of falling into a local optimum, which can be improved on in future research. However, the PSO algorithm has more advantages in optimizing models with multiple free parameters than the LM algorithm. For example, the PSO algorithm is significantly better in terms of convergence speed and fitting accuracy. Satisfactory multi-parameter optimization can remove the dependence on pre-characterization and also prevent the measurement error caused by pre-characterization failure.

## 4. Conclusions

This paper proposes a concentration retrieval technique based on the PSO algorithm, which is used for a calibration-free wavelength modulation spectroscopy system. Contrasted with the LM-based technique, the retrieval of gas concentrations by this technique is weakly dependent on the pre-characterization of the laser tuning parameters. The key parameters affecting the performance of the PSO technique are analyzed in depth. Furthermore, we comprehensively evaluate the performance of both the PSO and LM techniques through systematic testing and analysis under different conditions. The results indicate that the relative errors of best-fit parameters *i*_1_, *i*_2_, and *ψ*_1_ predicted by the LM technique exceed 5%, but all predicted by the PSO technique are less than 1%. The convergence speed of the PSO technique was about 63 times faster than the LM technique when the fitting accuracy remained the same. All these results prove that the PSO technique outperforms the LM technique in terms of convergence time and error for the model with multiple free parameters. The results of fitting the 300–500 ppm concentration spectra and the Gaussian white noise-containing spectra well demonstrate the adaptability and noise resistance of the technique.

## Figures and Tables

**Table 1:** Summary of spectral parameters.

Symbol	Quantity	Value
*m*	modulation index	1.5
*L*	absorption path length	20 cm
*ψ*_1_	phase shift between FM and linear IM	0.6 π rad
*ψ*_2_	phase shift between FM and nonlinear IM	0.5 π rad
	isotopologue	^12^C_2_H_2_
*ν_c_*	line-center frequency of transition	6523.8792 cm^−1^
Δ*ν_c_*/2	half width at half-maximum	0.0777 cm^−1^
*S*	line strength	1.035 × 10^−20^ cm/mol
*T*	arbitrary temperature	296 K
*P*	total pressure	1 atm
*i*_1_	linear IM depth at line-center frequency	0.15
*i*_2_	nonlinear IM depth at line-center frequency	3 × 10^−3^

**Table 2:** Parameter value ranges of the PSO technique.

Number of Particles: 1000	
Free Parameters	Region of Parameters
*m* [cm^−1^]	1.0–2.0
*c* [ppmv]	390.6–488.3
*i*_1_	0.08–0.35
*i*_2_	0–0.007
*ψ*_1_ [π rad]	0–1.0
*ψ*_2_ [π rad]	0–1.0

**Table 3:** The concentration and laser parameters predicted by the PSO technique with a convergence time of 5 s.

Free Parameters	Expected Value	Predicted by the PSO Technique	Relative Errors
*m* [cm^−1^]	1.500	1.500	0.00
*c* [ppmv]	400.0	400.5	0.13%
*i*_1_	0.150	0.149	0.67%
*i*_2_	0.003	0.003	0.00
*ψ*_1_ [π rad]	0.600	0.601	0.17%
*ψ*_2_ [π rad]	0.500	0.500	0.00

**Table 4:** Initial parameter value of the LM technique.

Free Parameters	Initial Parameter Value
*m* [cm^−1^]	1.4
*c* [ppmv]	390.6
*i*_1_	0.15
*i*_2_	0.003
*ψ*_1_ [π rad]	0.6
*ψ*_2_ [π rad]	0.5

**Table 5:** The concentration and laser parameters predicted by the LM technique with a convergence time of 100 s.

Free Parameters	Expected Value	Predicted by the LM Technique	Relative Errors
*m* [cm^−1^]	1.500	1.529	1.93%
*c* [ppmv]	400.0	410.7	2.68%
*i*_1_	0.150	0.1677	11.8%
*i*_2_	0.003	0.0034	13.3%
*ψ*_1_ [π rad]	0.600	0.647	7.83%
*ψ*_2_ [π rad]	0.500	0.487	2.60%

## Data Availability

Data underlying the results presented in this paper are not publicly available at this time but may be obtained from the authors upon reasonable request.
